# Preliminary experience with motorized distraction for tibial lengthening

**DOI:** 10.1007/s11751-014-0191-1

**Published:** 2014-03-15

**Authors:** Adam S. Bright, John E. Herzenberg, Dror Paley, Ian Weiner, Rolf D. Burghardt

**Affiliations:** 1 1950 Arlington Street, #111, Sarasota, FL 34242 USA; 2 International Center for Limb Lengthening, Rubin Institute for Advanced Orthopedics, Sinai Hospital of Baltimore, 2401 West Belvedere Avenue, Baltimore, MD 21215 USA; 3 Paley Advanced Limb Lengthening Institute, 901 45th Street; Kimmel Building, West Palm Beach, FL 33407 USA; 4 2700 Quarry Lake Drive Suite 300, Baltimore, MD 21209 USA; 5 Orthopaedic Department, Helios-Endo Klinik Hamburg, Hamburg, Germany

**Keywords:** Bone lengthening, Limb length discrepancy, Ilizarov, External fixator, Motorized distraction, Distraction osteogenesis, Tibia

## Abstract

Limb lengthening by callus distraction is commonly performed with the use of external fixation. Lengthening is routinely performed by the patient through small increments throughout the course of a day. Ilizarov has shown that both the rate and frequency of distraction are important factors in the quality of osteogenesis. We report the effect of motorized high frequency distraction for tibial lengthening in comparison with manual low-frequency distraction at the same rate. Manual distraction (0.25 mm four times a day) in a group containing 43 tibiae was compared with motorized distraction (1/1,440 mm 1,400 times a day) in a group containing 27 tibiae. There was no significant difference in time to union or in the incidence of complications.

## Introduction

Ilizarov was an innovator in the field of limb lengthening and published several classic works defining the key principles of limb lengthening [1, 2]. In a canine study, he performed tibial lengthening and analyzed factors influencing the healing rate in distraction osteogenesis, including the rate and frequency of distraction [2]. Using an experimental motorized distractor on dog tibiae, Ilizarov [2] studied the radiographic and histologic effects of different frequencies of distraction. He reported that bone healing was best in the group with motorized (1/60th mm, 60 times per day) distractors compared with the more standard rhythm (1/4 mm, 4 times per day). Korzinek et al. [3] also reported that bone regeneration in canines was greater with motorized distraction. However, Welch et al. [4] reported no difference between manual and motorized distraction in goats. Kreitz et al. [5] found no difference in the four-point bending strength of motorized lengthenings in sheep. The motorized lengthenings produced denser, more organized bone but with a smaller quantity, resulting in no difference in mechanical testing. Wiltfang et al. [6] claimed that the rhythm of distraction has a significant influence on bone regeneration in an animal study. They performed distraction osteogenesis of the mandible in minipigs, utilizing a microhydraulic cylinder to perform continuous distraction. They were unable to show any histologic difference, although they reported accelerated bone healing when measurements were obtained with ultrasonography and electron microscopy.

To date, no comparative study of motorized distraction in humans has been published. The purpose of the current study was to evaluate our initial clinical experience with motorized distraction (at a rate of 1/1,440 mm 1,440 times per day) compared with manual distraction (at a rate of 0.25 mm four times per day).

## Materials and methods

**Fig. 1:**
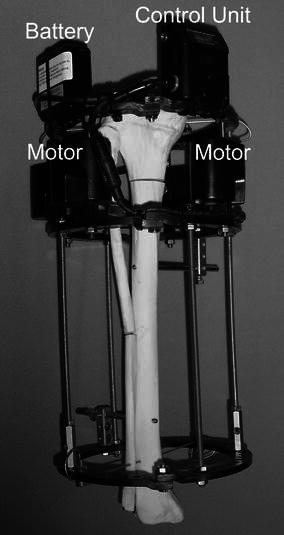
The original motorized distraction device (Autogenesis, Inc., Baltimore, MD) on an Ilizarov ring fixator on a saw-bone model. This device consists of four motors, a battery pack and a control unit

This feasibility study had institutional review board approval. During a 2-year period, 26 patients underwent 27 single-level proximal tibial lengthenings with motorized distraction at a rate of 1 mm per day and a rhythm of 1/1,440 mm, 1,440 times a day (Autogenesis, Inc., Baltimore, MD) (Fig. 1). The decision to use motorized instead of regular lengthening struts was made based on published and theoretical advantages in bone healing and patient preference. The patients with motorized distractors were informed of the theoretical advantages in bone healing with motorized versus manual distractors. Comparison was made to a manual lengthening group (43 tibiae in 40 patients), which had been treated in the 3 years prior (historical cohort). The manual lengthening group underwent proximal tibial lengthening at a rate of 1/4 mm of distraction 4 times per day (1 mm per day). The average age of the patients was 20 years for both groups.

All proximal tibial osteotomies were done either by Gigli saw or by osteotome percutaneously. A previous study showed no difference in healing of proximal tibial metaphyseal osteotomies using either technique [7].

The Ilizarov device was used for all lengthenings. The date of frame application, date of diagnosis of bone union and length of distraction were recorded. The objective radiographic criteria used to determine bone union were absence of the fibrous interzone and the presence of three of four (anterior, posterior, medial and lateral) well-defined cortices on anteroposterior and lateral radiographs [8]. The interobserver error of measuring bone union was determined by ensuring the radiographs were reviewed independently by two observers. Because the interobserver error was significant (*p* = 0.01), one observer was used for all tibiae. Complications related to motor dysfunction during lengthening or union time (fracture or bending of the regenerate bone) were recorded. Data were analyzed using the Statistical Analysis System, version 6.0. Wilcoxon scores of rank sums were used to determine the effects of distraction length, patient age and motorized distraction. Regression analysis to control for the effect of length of distraction was conducted to examine the effects of other variables. The influence of motorized distraction on union time then was analyzed using a paired t test.

## Results

The difference in age distribution for patients in both groups was not statistically significant (*p* = 0.53). Twenty-six tibial lengthenings (60 %) in 24 patients were aged 19 years or younger in the manual group in comparison with 17 tibial lengthenings (40 %) in 10 patients in the motorized group. Seventeen tibial lengthenings (63 %) were performed in 16 patients aged 20 years and older in the manual group in comparison with 10 tibial lengthenings (37 %) in 16 patients in the motorized group.

The mean distraction length was 4.2 cm (range 0.3–11.1 cm; one case of 0.3 cm lengthening was an angular deformity correction that actually lengthened much more on the concave side but measurements were made on the shorter, convex side) for the manual group (4.1 cm for patients age 19 years or younger and 4.2 cm for patients 20 years or older). The average lengthening was 3.1 cm (range 1.0–7.4 cm) for the motorized group (3.8 cm for patients age 19 years or younger and 2.3 cm for patients 20 years or older). Although the amount of lengthening was slightly less in cases of motorized distraction, this was not statistically significant (*p* = 0.09).

Union time was dependent on distraction length for patients in both groups. There was no significant difference in time to union (*p* = 0.25) when comparing manual versus motorized lengthenings for patients younger than 19 years. For all ages combined, there was no significant difference in the time to union between the motorized group and the manual group (*p* = 0.5).

Patients aged 20 years or older in both groups experienced a longer time to union compared with patients 19 years or younger in both the manual and motorized groups. There was a trend for motorized lengthenings to take longer when the distraction gap was less than 5 cm for patients older than 19 years, but this was not statistically significant (*p* = 0.86).

There was no significant difference in fracture rate; there were two fractures in the manual group (5 %) and one fracture in the motorized group (4 %). There were four different mechanical failures in the motorized group: dead batteries, a bent plug, a bent rod and a motor that failed because of torque. The unexpected battery failures were caused by excessively high chlorine levels in the rehabilitation facility swimming pool that dissolved the neoprene seals. All of these problems were diagnosed quickly, the failures were repaired and the lengthening procedures were continued.

## Discussion

Fischgrund et al. [8] studied bone lengthening and reported that patient age, distraction length and the bone segment being lengthened all affect time to union. This study affirms that age and distraction length have the same effect on time to union. However, our data suggest that motorized distraction at 1 mm per day in 1,440 steps (one step a minute) does not improve time to union significantly nor does it reduce complications of bone fracture when compared with a traditional manual distraction rate of 1 mm per day in four steps. These findings support those of the animal studies conducted by Welch et al. [4] and Kreitz et al. [5], but contradict studies conducted by Ilizarov [2] and Korzinek et al. [3]. The latter two studies looked at the histologic differences, whereas the former two compared strength of bone formed in the distraction gap. Kreitz et al. [5] showed that the improved organization of regenerated bone from motorized distraction compensates for smaller volumes of bone formation.

There are several limitations in this work. Only metaphyseal lengthenings (which heal faster than diaphyseal lengthenings) were studied. The main outcome was time to bone union, and potential benefits to soft tissue, e.g., muscle, nerve or cartilage were not recorded [9]. Nakamura et al. [10] showed histologic evidence of tibial articular cartilage damage in rabbits that had undergone tibial lengthenings of 1 mm per day in 120 steps per day. They found significantly less damage in motorized lengthening when compared with their manual group that was distracted at 1 mm per day in two steps per day.

This study did not investigate the level of pain during lengthening or narcotic use during the treatment period, and further investigation into these two parameters may reveal possible differences.

This study was retrospective and based on a historical cohort. Observer bias may have been introduced from the measurements obtained from X-rays (the motors are seen on radiographs) and the subjective nature of determining union times. These limitations would be addressed by a randomized prospective design.

## Conclusion

**Fig. 2:**
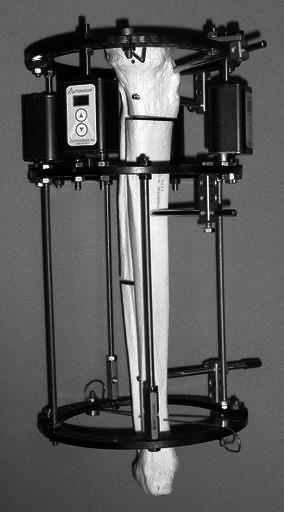
The new motorized distraction device (Autogenesis, Inc., Baltimore, MD) on an Ilizarov ring fixator on a saw-bone model. In this even smaller device, the four motors already include the battery and control unit

This preliminary investigation into use of motorized distraction shows no significant difference in time to bone union. The potential benefits of improved compliance and patient convenience from using such devices need to be weighed against the increased cost, weight (1 kg for the initial device used in this study) and potential for mechanical breakdown. There may be a case made for pediatric patients who may show anxiety over use of wrenches on manual distraction struts and for those select adult patients where compliance is a concern. In comparison with the bulky early devices used for this study, there are newer, smaller and lighter commercially available versions which might overcome some of the physical disadvantages (Fig. 2).
